# Prediction of Episodic Memory With Multiomics Scores

**DOI:** 10.1016/j.bpsgos.2025.100607

**Published:** 2025-09-06

**Authors:** Anni L.K. Malmberg, Matti Pirinen, Johannes Kettunen, Katri Räikkönen, Johan G. Eriksson, Jari Lahti

**Affiliations:** a Department of Psychology, University of Helsinki, Helsinki, Finland; b Department of Mathematics and Statistics, University of Helsinki, Helsinki, Finland; c Institute for Molecular Medicine Finland, Helsinki Institute of Life Science, University of Helsinki, Helsinki, Finland; d Department of Public Health, University of Helsinki, Helsinki, Finland; e Systems Epidemiology, Research Unit of Population Health, Faculty of Medicine, University of Oulu, Oulu, Finland; f Biocenter Oulu, University of Oulu, Oulu, Finland; g Department of Obstetrics and Gynecology, Helsinki University Hospital, Helsinki, Finland; h Folkhälsan Research Centre, Helsinki, Finland; i Department of Obstetrics and Gynaecology and Human Potential Translational Research Programme, Yong Loo Lin School of Medicine, National University of Singapore, Singapore; j Department of General Practice and Primary Health Care, University of Helsinki, Helsinki, Finland

**Keywords:** Episodic memory, Genome-wide association analysis, Metabolome-wide association analysis, Multiomics, Neurocognition, Prediction

## Abstract

**Background:**

Episodic memory (EM) refers to the ability to encode and recall events—a vital cognitive function for healthy cognitive aging and an endophenotype for dementia.

**Methods:**

Using genome- and metabolome-wide least absolute shrinkage and selection operator (LASSO) analysis, we developed polygenic (LASSO-PRS) and metabolic risk scores (MRS) in ∼68.5-year-old individuals (*n* = 897). We also applied the Bayesian regression method PRS-CS to an external genome-wide meta-analysis (GWAMA, *N* = 29,785, age > 18 years) to derive another PRS (GWAMA-PRS). We assessed incremental variances (*R*^2^) in EM explained by the PRSs and MRS separately and in combination beyond the Cardiovascular Risk Factors, Aging, and Incidence of Dementia (CAIDE) score in 104 independent ∼68.5-year-old individuals. Finally, we validated the PRSs in 2 independent pediatric cohorts (*N* = 309, age = ∼11.9 years; *N* = 443, age = ∼8.6 years).

**Results:**

In the independent sample of ∼68.5-year-old individuals, compared with CAIDE score alone, accounting additionally for either MRS, LASSO-PRS, or GWAMA-PRS increased *R*^2^ by 1.6, 5.6, and 4.5 percentage points (pp), respectively, while accounting additionally for MRS + LASSO-PRS or MRS + GWAMA-PRS increased *R*^2^ by 7.8 and 6.4 pp, respectively. Both LASSO-PRS (all false discovery rate [FDR]–adjusted *p* values = .01–.03) and GWAMA-PRS (all FDR-adjusted *p* values = .03) were significantly associated with EM in all models, while the CAIDE score and MRS were not (all FDR-adjusted *p* values > .05). PRSs were not associated with EM in the pediatric cohorts (all FDR-adjusted *p* values > .05).

**Conclusions:**

Genomics added predictive value to EM beyond epidemiological risk factors in adults, but the same was not observed with metabolomics. Adult-derived PRSs did not predict EM in children.

## Plain Language Summary

Episodic memory—the ability to encode and recall events over a short time interval—is important for healthy aging and is often affected early in dementia. In our study, genomic markers enhanced prediction of memory performance beyond traditional risk factors, such as age and lifestyle, and metabolomic markers in individuals without dementia. This suggests that genomic markers could support risk stratification of dementia before its onset, allowing for timely support and preventive strategies.

Episodic memory (EM) refers to the ability to encode and retrieve events over a short time interval (1), a cognitive process that is vital in daily life and affected by normal aging. Better EM is associated with higher education (2) and reduced risk of cognitive impairment (3) and decline (4). EM is an endophenotype for dementia (5). EM tests, e.g., wordlist tests, discriminate individuals with Alzheimer’s disease (AD), the most common cause of dementia, from healthy control individuals rather well (6). Early identification of individuals at risk for AD is crucial as emerging evidence suggests that interventions for AD should be initiated in the early stages of the neuropathological processes leading to AD to have the potential for effectiveness (7, 8, 9). Several studies have utilized traditional epidemiological markers and biomarkers in the early identification and prediction of AD and other dementias.

The Cardiovascular Risk Factors, Aging, and Incidence of Dementia (CAIDE) score based on cardiovascular risk factors—age, male sex, low education, physical inactivity, obesity, hypertension, and hypercholesterolemia—predicted later-life dementia over 20- and 36-year follow-ups in middle-age individuals of Finnish (*N* = 1409) (10) and other ethnicities (*N* = 9480) (11), respectively. The CAIDE score has also been associated with other cognitive outcomes (12,13), including poorer EM (12).

Polygenic risk scores (PRSs), which utilize a large part of common genomic variation, have been shown to predict the risk of AD (14, 15, 16, 17), vascular dementia (16) and all-cause dementia (16,18), and age of onset of AD (17), and therefore could aid in early identification of dementias. PRSs have also been shown to predict cognitive abilities, including EM (19,20), verbal-numerical reasoning (19,20), nonverbal reasoning (20), perceptual-motor speed (20), processing speed (19,20), executive functioning (20,21), general cognitive function (22,23), intelligence (24,25), and educational attainment (19,23,26). However, the performance of PRSs tends to be population specific (27); for example, 2 recent studies (19,20) found that the PRSs for EM explained only 0% to 2% of the variation in the outcome when validated in independent cohorts. In addition, it is widely acknowledged that complex traits, such as cognitive outcomes, may also be characterized by other molecular markers. For example, in recent years, metabolomics has been used in the prediction of decline in cognitive performance [e.g., in verbal EM (28)] (28,29), as well as of cognitive impairment (30,31), AD (28), and other dementias (32,33).

Integrating multiple omics predictors may further enhance prediction of clinical outcomes. For AD, integration of gene expression data with methylomics (34) and genomics (35) has added predictive value over single omics models. Two recent studies (36,37) have shown that in comparison with models including only disease-specific conventional epidemiological or clinical risk scores, integration of the conventional risk scores and 2 different omics [genomics and gut microbiome (36) or genomics and metabolome (37)] together improved prediction of 4 diseases (including AD) in Liu *et al.* (36) and 8 of 9 diseases in Barrett *et al.* (37).

We are not aware of any studies integrating multiple omics in the prediction of EM, and in general, prediction models for AD and other dementias are more common. A prediction model for EM could enable the identification of individuals at risk for dementia before diagnostic criteria are satisfied. Because both genomics and metabolomics are associated with neurocognitive health, but genomics captures life-long predispositions while metabolomics reflects current systemic physiology, combining these omics may provide complementary insights for prediction of EM. In addition, because general cognitive ability in late adulthood and childhood shows high genetic correlation (38), an adult-derived PRS for cognitive abilities could be predictive also in children.

Therefore, we first used genome- and metabolome-wide least absolute shrinkage and selection operator (LASSO) analysis to develop polygenic (LASSO-PRS) and metabolic risk scores (MRS) in ∼68.5-year-old individuals (*n* = 897). We then evaluated the predictive performances of the CAIDE, PRS, and MRS for EM in independent ∼68.5-year-old individuals (*n* = 104). Our key focus was to assess whether the PRS and MRS separately or in combination improved prediction of EM over and above the CAIDE score. In addition, we tested whether the PRS enhanced prediction of EM beyond epidemiological markers in 2 independent child cohorts (*N* = 309, age = ∼11.9 years; *N* = 443, age = ∼8.6 years). In contrast to, e.g., standard genome-wide association study (GWAS), LASSO models predictors jointly, reducing noise and controlling multicollinearity and consequently increasing statistical power. Therefore, for comparison, we developed another PRS based on an external, standard genome-wide association meta-analysis (GWAMA) (GWAMA-PRS) of verbal EM by Lahti *et al.* (39), which again gained power from substantially larger sample sizes (average *N*/cohort ≈ 1278, total *N* = 29,785) than our genome-wide LASSO. While LASSO stabilized effect size estimates through penalization, for GWAMA-PRS, posterior effect sizes were inferred via PRS-CS (40)—a Bayesian framework with continuous shrinkage priors informed by local multicollinearity patterns.

## Methods and Materials

### Data

#### Study Participants

Participants are from 3 Finnish cohorts: the HBCS (Helsinki Birth Cohort Study) (41,42), PREDO (Prediction and Prevention of Preeclampsia and Intrauterine Growth Restriction) (43), and GLAKU (Glycyrrhizin in Licorice Study) (44), comprising individuals born from 1934 to 1944, from 2006 to 2010, and in 1998, respectively. Descriptions of cohort designs are provided in the Supplement.

All HBCS participants in our study sample (*N* = 1001) had data on cognitive functioning assessed between 2006 and 2011 and on the dementia risk factors included in the CAIDE score. Of these participants, 846 donated blood for genotype assaying in clinical examinations in 2001 to 2004, and 810 donated fasting blood for assessment of metabolomics profiles between 2006 and 2008, on average 2.2 (SD = 0.6) years before cognitive assessment, and 655 participants donated blood for both.

We compared our total HBCS study sample (*N* = 1001) to the participants who were not in our study (*N* = 1002) on the CAIDE score and its components (age, sex, education in years, body mass index [BMI], systolic blood pressure in mmHg, total cholesterol in mmol/L, and physical inactivity). Participants in our study were more often women than men (*p* value = .0006); had higher education (*p* value = .006); and showed lower systolic blood pressure (*p* value = .0002), BMI (*p* value = .0009), and CAIDE scores (*p* value = .019) than participants not in our study (Table S1). Age at cognitive assessment, total cholesterol, and physical inactivity did not differ between the 2 groups.

In PREDO and GLAKU, data on cognitive assessments, along with child covariates—child’s age at cognitive assessment, sex, and maternal education—were available for 443 ∼8.6-year-old children and 309 ∼11.9-year-old children. In PREDO, children in our study (*N* = 443) were younger (*p* value = .002), and mothers of the children were more highly educated (*p* value = 1.43 × 10^−6^) than mothers of those who were not in our study (*N* = 725) (Table S2), while in GLAKU there were no attrition biases in the covariates between those who were (*N* = 309) and were not (*N* = 611) in our study (Table S3).

Written informed consent was provided by all HBCS participants and the parents of children from the GLAKU and PREDO cohorts. The study protocols were approved by the Institutional Review Board of the National Public Health Institute and Ethics Committees of the Hospital District of Helsinki and Uusimaa (HBCS), Ethics Committees of the Helsinki and Uusimaa Hospital District (PREDO), and Ethics Committees of the City of the Helsinki and Uusimaa Hospital District (GLAKU).

#### EM Assessments

To assess EM in the HBCS sample, we used the Consortium to Establish a Registry for Alzheimer’s Disease-Word List Memory test (CERAD WLM) (45). It is a free recall memory test that assesses learning ability for new verbal information. A 10-item word list was presented over 3 trials (at the rate of 1 every 2 seconds) with a different word order in each trial. The participant was instructed to read each word aloud as it was presented and then asked to recall as many words as possible. The maximum number of correct responses was 10 for each trial, with a trial total of 30.

In both PREDO and GLAKU, we used subtests from the Memory and Learning domain from the NEPSY Second Edition (46). For PREDO, the subtest was Memory for Faces with delayed recall, which assesses long-term facial memory. For GLAKU, we used the total scores for the subtests Narrative Memory, in which the participant is asked to recall a narrative freely, and Memory for Names, the first part of which assesses learning of names related to facial pictures over 3 trials (46).

#### Genotyping

DNA was extracted from whole blood in HBCS, cord blood in PREDO, and whole blood and saliva in GLAKU. All cohorts followed standard protocols for genotyping, quality control, and imputation (see details in the Supplement).

#### Quantification of Metabolomics in HBCS

Our study included 137 fasting blood metabolic measures from the Nightingale nuclear magnetic resonance metabolomics (47) platform (Supplement and Table S4).

### Statistical Analyses

Details of the methodology applied in constructing the CAIDE score, LASSO-PRS, MRS, and GWAMA-PRS are provided in the Supplement. Briefly, we first constructed the CAIDE score, as suggested previously (10), in the HBCS participants (*N* = 1001). A higher score indicated more dementia risk factors. Second, a common test sample (*n* = 104) was randomly selected from participants with complete data (*n* = 655) for both genomic and metabolomic analyses. Third, in the remaining nontest participants (total *n* = 897), using LASSO (48, 49, 50), we analyzed associations of genome-wide genetic variants (LASSO-GWAS; *n* = 742) and metabolomic measures (LASSO-MWAS; *n* = 706) separately with EM. Penalization parameters were optimized via a single training validation split (LASSO-GWAS) or cross-validation (LASSO-MWAS) as recommended by each method’s documentation (49,50). Both LASSO models were adjusted for CAIDE score and age at EM assessment. In addition, the LASSO-GWAS was adjusted for genetic ancestry, and the LASSO-MWAS was adjusted for the time interval between blood donation for metabolome profiling and EM assessment. The final LASSO-PRS and MRS were constructed as weighted sums of the selected predictors using LASSO-estimated effect sizes as weights.

Fourth, we derived another PRS by applying the Bayesian regression tool PRS-CS (40) to meta-analysis of standard univariate GWASs (GWAMA) on EM previously conducted by our group (39) in the CHARGE Consortium (including HBCS). The cohorts in this GWAMA used assessments of verbal learning comparable to the CERAD WLM used in the HBCS, although the number of learning trials and words varied slightly across the cohorts. For the current study, we recomputed the GWAMA excluding HBCS participants (remaining total *N* = 29,785).

Fifth, to predict EM in the HBCS test sample (*n* = 104), we fitted standard linear regression models, each including either one of the risk scores—CAIDE, MRS, LASSO-PRS, or GWAMA-PRS—or one of the PRSs combined with the CAIDE score and/or MRS, while adjusting for the non-CAIDE (background) covariates. For each pair of nested models, where one of the models served as baseline and the other had been extended with additional risk score(s), we quantified the incremental proportion of explained variance (*R*^2^) as the extended model’s *R*^2^ minus the baseline *R*^2^. Because incremental *R*^2^ is always positive, statistically significant model improvement was inferred through adjusted *R*^2^, predictor-specific *p* values, and changes in Akaike information criterion (AIC) and Bayesian information criterion (BIC) values between each extended and baseline model. We compared model performance between the LASSO-PRS and GWAMA-PRS models. As a sensitivity analysis, we repeated the same analyses after excluding 3 participants with stroke before EM assessment (*n* = 101). We performed false discovery rate (FDR) correction for multiple testing within each layer of data—CAIDE as the epidemiological predictor, the MRS for metabolomics, and the PRSs for genomics—across all models and both HBCS-derived test datasets, using the Benjamini-Hochberg method [R’s p.adjust() function] (51). To control possible inflation of significance of the PRS models relative to the CAIDE and MRS models, we applied an additional FDR correction separately to each PRS. Under each correction approach, predictors with FDR-adjusted *p* values < .05 were considered statistically significant. Finally, we tested whether adult-driven PRSs associated with EM in children (PREDO and GLAKU) using standard linear regression. As the CAIDE score is not applicable in children, we compared the model including child’s age at EM assessment, sex, and maternal education with models extended with either PRSs. In all test datasets, we standardized (mean = 0 and SD = 1) both PRSs and the MRS to estimate their effect sizes in the regression models per 1 SD.

All analyses were conducted in R version 4.0.0, except GWAMA, which was conducted with METAL (52), and GWAMA-PRS, which was constructed with the PRS-CS software (40).

We applied the snpXplorer AnnotateMe tool (53) to annotate both the variants selected for LASSO-PRS and the statistically significant variants (meta-analysis *p* value < 5 × 10^−8^) included in GWAMA-PRS.

## Results

In the HBCS nontest sample, LASSO selected 274 genetic variants for the LASSO-PRS (Table S6) and 15 metabolic measures for the MRS (Table S5). The GWAMA-PRS included 1,083,016 single nucleotide polymorphisms for HBCS, 1,097,319 for GLAKU, and 1,082,409 for PREDO, and each included 12 (top) variants with a meta-analysis *p* value < 5 × 10^−8^ (Table S7). Based on functional annotation, some of the LASSO-PRS variants (Tables S8–S11) and all the 12 top GWAMA-PRS variants (Tables S12–S15) showed potential as markers for neurocognition. In addition, some metabolic measures demonstrated biological plausibility (Table S5).

### Prediction of EM With MRS, LASSO- and GWAMA-Based PRSs in the HBCS Test Sample

**Figure 1:**
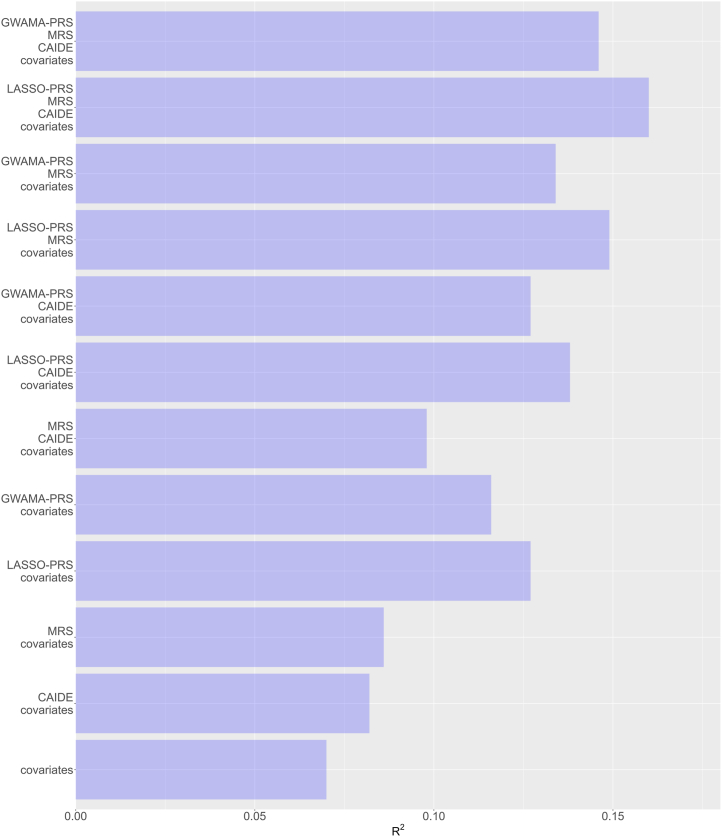
Proportions of variances (*R*^2^) in EM explained by traditional epidemiological risk factors for dementia (CAIDE score), MRS, LASSO-PRS, GWAMA-PRS, and their combinations after adjusting for the background covariates (age at EM assessment, time interval between blood donation for metabolome profiling and EM assessment, genetic ancestry) in the HBCS test sample (*n* = 104). After FDR correction for multiple testing within each data layer (CAIDE, MRS, or PRS separately) across all models and the 2 independent Helsinki Birth Cohort Study–derived test datasets—the current one including 3 participants diagnosed with a stroke before EM assessment and the other excluding them (not shown here)—FDR-adjusted *p* values for the CAIDE score, MRS, LASSO-PRS, and GWAMA-PRS were 0.28, 0.20, 0.03, and 0.03, respectively, in every model. After FDR correction separately to each PRS, the adjusted *p* values were 0.01 for the LASSO-PRS and 0.03 for the GWAMA-PRS in every model. CAIDE, Cardiovascular Risk Factors, Aging, and Incidence of Dementia; EM, episodic memory; FDR, false discovery rate; GWAMA, genome-wide association meta-analysis; LASSO, least absolute shrinkage and selection operator; MRS, metabolic risk score; PRS, polygenic risk score.

All standard regression results for the CAIDE score, MRS, LASSO-PRS, and GWAMA-PRS on EM in the HBCS test sample are represented in Table S16, and the explained variances (*R*^2^) of each model are shown in Figure 1. In the HBCS test sample (*n* = 104), compared with the CAIDE + background covariates model, adding the MRS, LASSO-PRS, or GWAMA-PRS increased *R*^2^ by 1.6, 5.6, or 4.5 percentage points (pp), respectively, while adding the LASSO-PRS + MRS or the GWAMA-PRS + MRS increased *R*^2^ by 7.8 or 6.4 pp, respectively.

The CAIDE + background covariates model explained *R*^2^ by 1.2 pp more than the background covariates alone. In other model comparisons, depending on the baseline, adding individual predictors increased the *R*^2^ by 1.6 to 2.2, 5.7 to 6.3, or 4.6 to 4.9 pp for the MRS, LASSO-PRS, and GWAMA-PRS, respectively. Relative to the background covariates alone, accounting additionally for the CAIDE score plus either MRS, LASSO-PRS, GWAMA-PRS, LASSO-PRS + MRS, or GWAMA-PRS + MRS, yielded respective incremental *R*^2^s of 2.8, 6.9, 5.7, 9.0, or 7.6 pp. Finally, combining the MRS + background covariates with the LASSO-PRS or GWAMA-PRS increased *R*^2^ by 7.9 or 6.5 pp, respectively, beyond the background covariates alone.

Consistently across both FDR correction approaches, both the LASSO-PRS (after within-layer correction, all FDR-adjusted *p* values = .03; after correction for LASSO-PRS models only, all FDR-adjusted *p* values = .01) and the GWAMA-PRS (all FDR-adjusted *p* values = .03) were significantly associated with EM in all models, whereas neither the CAIDE score (all FDR-adjusted *p* values = .28) nor the MRS (all FDR-adjusted *p* values = .20) was significant. All significant findings remained after excluding the 3 participants with stroke (Table S16). Including either PRS always improved model fit, as indicated by their FDR-adjusted *p* values, higher adjusted *R*^2^, and lower AIC and BIC values. LASSO-PRS models consistently showed slightly higher incremental *R*^2^s than GWAMA-PRS models.

### Prediction of EM With LASSO- and GWAMA-Based PRSs in Children

In GLAKU and PREDO, the LASSO-PRS included 255 (93.0%) and 259 (94.5%) of the variants. Neither the LASSO-PRS nor the GWAMA-PRS was significantly associated with any measures of EM in GLAKU or in PREDO (all FDR-adjusted *p* values > .05) (Tables S17 and S18).

## Discussion

While some studies have examined whether combining multiple levels of omics data improves prediction of AD (34, 35, 36, 37) and other diseases (36,37), to the best of our knowledge, such an approach has not been applied to EM before. In this study, we investigated whether traditional epidemiological markers for dementia (CAIDE score) and genomic (LASSO-PRS and GWAMA-PRS) and metabolomic (MRS) markers predicted EM in late adulthood (∼68.5 years). The key objective was to assess whether the genomic or metabolomic markers, separately or in combination, improved prediction beyond the traditional markers alone. We also examined whether the adult-derived genomic markers predicted EM in children.

The CAIDE score, which comprises traditional dementia risk factors, was not associated with EM in late adulthood. One possible explanation for this is the limited size (*n* = 104) of the HBCS test sample. In addition, our total study sample (*N* = 1001) had fewer dementia risk factors—and consequently lower CAIDE scores—than the group of individuals excluded. On the other hand, previous studies have shown inconsistent results when validating the CAIDE against dementia or cognitive outcomes; its ability to predict dementia has ranged from very high (11) to moderate (54) or even poor (55,56). CAIDE has also been linked to poorer EM (12), executive functioning (12), processing speed (12), and general cognition (12,13). Moreover, some studies have found no association of CAIDE with memory (57,58), while CAIDE has been associated with greater cognitive decline (57) or impairment (58) in certain other cognitive abilities. Furthermore, as CAIDE was originally developed in a midlife cohort (mean age = 53.4 years), its applicability may be reduced in older cohorts, where the impact of risk factors such as BMI and cholesterol levels may shift with age (55,56).

Because both PRSs associated with EM in the HBCS test sample under both multiple-testing correction approaches—despite none of the participants having dementia and the associations remaining significant after excluding those with stroke before EM assessment—these findings may not be due to evident neuropathologies. Across all baseline models, adding either of the PRSs improved the model significantly, with the LASSO-PRS slightly outperforming the GWAMA-PRS.

The proportions of variances in EM explained by PRSs are higher than those reported by Davies *et al.* (19) and de la Fuente *et al.* (20). In their studies, PRSs for EM, constructed using the UK Biobank (UKBB) sample, explained only 0% to 2% of the variance in independent cohorts, despite much larger training (*N* = 112,067–331,679) and test sample sizes (*N* = 1005–19,994) than ours. However, the small proportion of variance explained may be due to limited reliability of the pairs-matching test in the UKBB (59,60), as indicated by a low test-retest correlation (0.15) also in Davies *et al.* (19).

The LASSO-PRS and GWAMA-PRS constructed in ∼68.5- and >18-year-old individuals, respectively, were significantly associated with EM in the HBCS test sample but not in children of the PREDO and GLAKU cohorts. Possible reasons for this include age-related developmental differences in cognitive functions (61), the potential moderation of genetic effects on EM by age (62), different EM assessments across all 3 cohorts (verbal EM in HBCS and visual EM in PREDO), and missing (5.5%–7.0%) LASSO-PRS variants, together with small sample sizes in the PREDO and GLAKU sample.

The MRS was not associated with EM in the HBCS test sample. This was surprising as some metabolic markers have shown relevance to cognition-related outcomes (Table S5). For example, apolipoprotein A-I has previously been associated with reduced AD risk (63,64), possibly due to its roles in brain lipid regulation or anti-inflammatory pathways (63,64). Elevated urea has been consistently linked to AD (65), vascular dementia (66), dementia with Lewy bodies (67), Parkinson’s disease dementia (68), and Huntington’s disease (69,70), suggesting a shared underlying mechanism potentially involving, e.g., neurotoxicity or metabolic dysfunction (66,67,69). In addition, elevated citrate has been linked to higher risk of AD (71) and other dementias (33), and higher apolipoprotein B by apolipoprotein A-I correlates with lower educational level (72). However, some studies have not found associations of apolipoprotein A-I (73,74), citrate (74), or apolipoprotein B by apolipoprotein A-I (73,74) with dementias (73) or cognitive abilities (74). Our study, as well as some previous ones, might have lacked power to detect associations between certain metabolic measures and cognitive outcomes.

Both the genomic LASSO- and GWAMA-PRSs explained EM over and above the traditional risk factors for dementia as reflected in the CAIDE score and/or metabolomic markers in the MRS. This implies that the prediction models for EM should include genomic markers in addition to traditional dementia risk factors and that some of the genomic effects on neurocognition may be direct instead of being mediated by the traditional dementia risk factors or metabolomics. However, the current results do not support added value of combining genomics and metabolomics in prediction of EM, as the MRS showed no significant association, although insufficient statistical power could be a partial explanation for this. Partially contrary to our findings, in the only published study known to us comparing the predictabilities of trait-specific clinical risk scores, PRSs, and MRSs both individually and in different combinations for cognition-related outcomes (37), both a PRS and an MRS predicted AD, and both the clinical score and the MRS predicted vascular and other dementias, and none of the scores added substantial predictive value over or above one another for the respective outcomes. Full models with all levels of data were not applicable for these outcomes as the study lacked a clinical risk score for AD and a PRS for vascular and other dementias (37).

We want to highlight several strengths of the current study. First, the CERAD WLM test, which we used in the HBCS, is widely used in screening for dementia and provides a reliable measurement of EM in late adulthood, when the risk of dementias starts to increase. Second, we were able to integrate genomic and metabolomic data with assessment of EM in participants free of dementia and stroke. Third, because performance in verbal EM has been shown to decline years before the onset of mild cognitive impairment preceding AD (3), and the LASSO- and GWAMA-PRS predicted verbal EM in the independent HBCS test sample free from dementia, the PRSs may enable even earlier risk stratification. Unfortunately, we did not have enough dementia cases in the HBCS to validate our prediction models against dementia.

Fourth, by applying LASSO and PRS-CS, both of which shrink effect sizes of the predictors while accounting for correlations among them, we reduced noise, addressed multicollinearity, and stabilized effect size estimates. Additionally, the standard GWASs included in the external GWAMA had substantially larger sample sizes than our LASSO-GWAS on average. Together, these methodological strategies enhanced statistical efficiency and improved generalizability of the risk scores compared with the traditional univariate genome- and metabolome-wide association, relying on strict inclusion thresholds. Fifth, when examining predictive performance of the CAIDE score, MRS, and PRSs in the HBCS test datasets, we applied multiple-testing corrections both within each data layer (CAIDE, MRS, and PRSs) and—to control possible inflation of significance of the PRS models relative to the CAIDE and MRS models—for each PRS separately. Notably, both PRSs remained statistically significant under both correction approaches.

However, our study also has limitations. Our power to detect associations, especially in the HBCS test sample, may be limited due to the sample size. In PREDO and GLAKU, limited sample sizes impeded training PRS models in children and testing children-derived PRSs in adults. Moreover, because both PRSs were developed in adult cohorts of European ancestry, and the predictive utility of both PRSs was confined to adults, caution is warranted when extrapolating the PRS findings beyond populations of similar age and ancestry. In particular, some signals captured by the LASSO-PRS may be specific to certain populations or the method of EM assessment as LASSO-PRS slightly outperformed the GWAMA-PRS in the HBCS-derived independent sample. In addition, given the high polygenicity of EM (75), the number of variants selected for our LASSO-PRS remains low. Despite the fact that both of the PRSs improved prediction of EM, their predictive accuracies fall short of heritability estimates [30%–60%, as reported in twin studies (75)], and their utility in clinical prediction of dementia may be limited. These issues likely reflect insufficient GWAS sample sizes and have been commonly observed in studies linking PRSs with complex traits (76). Furthermore, the 2 PRS approaches had tradeoffs affecting biological interpretability: LASSO produced a sparse set of variants but not statistical significance values, while GWAMA-PRS (via PRS-CS) incorporated a dense set of variants but enabled inference based on GWAMA-derived *p* values. Nonetheless, functional annotation highlighted several LASSO-PRS variants and all top 12 GWAMA-PRS variants (*p* value < 5 × 10^−8^) as potential markers for neurocognition.

In addition, blood samples for assessment of metabolic profiles were drawn slightly before the EM assessment, possibly hindering our ability to find associations between the MRS and EM. However, we adjusted our analyses for the elapsed time difference. Finally, maximizing sample size through separate genome- and metabolome-wide analyses came at the cost of reduced insight into integrative associations. Limited sample size impeded joint modeling.

### Conclusions

Genomic variants may add predictive value for EM over and above traditional epidemiological dementia risk factors in late adulthood, but the same may not pertain to metabolomic measures. As the current adult-derived PRSs predicted EM in an independent, late adulthood, stroke- and dementia-free sample, the PRSs could support early risk stratification for dementia before the first neuropathological symptoms appear, thereby enabling targeted interventions to preserve cognitive abilities in individuals at risk. However, verifying this would require replication. Notably, the adult-derived PRSs did not predict EM in children, suggesting that the PRSs capture age-dependent biological pathways. Remaining important directions for future research include exploring the generalizability of a children-derived PRS for EM to adults, validating the prediction models against different types of dementia or other neuropathologies, and adding other data layers (e.g., proteomics, transcriptomics, epigenomics) and selecting integrative multiomics associations for prediction of EM.

## Supplementary Material

Supplemental Methods, Results, Figures S1–S2, and Tables S1–S3 and S7

Tables S4–S6 and S8–S18

Key Resources Table
