# Supplementary material for: Prediction of Episodic Memory With Multiomics Scores
Source: Biol Psychiatry Glob Open Sci. 2025 Sep 6;6(1):100607. doi: 10.1016/j.bpsgos.2025.100607 (PMC12554030; doi:10.1016/j.bpsgos.2025.100607)
Supplement: Supplemental Methods, Results, Figures S1–S2, and Tables S1–S3 and S7 [file mmc1.pdf]

SUPPLEMENTARY INFORMATION

Prediction of Episodic Memory With Multi-Omics Scores

Malmberg *et al.*

Table of Contents

Cohort Descriptions 3

Helsinki Birth Cohort Study (HBCS) 3

Prediction and Prevention of Preeclampsia and Intrauterine Growth Restriction (PREDO) ... 3

Glycyrrhizin in Licorice Study (GLAKU) 4

Supplementary Methods 5

Genotyping, Quality Control and Imputation 5

Quality control on the genotype data in HBCS 5

Quality control on the genotype data in PREDO 5

Quality control on the genotype data in GLAKU 6

Quantification of metabolomics in HBCS 6

Cardiovascular Risk Factors, Aging and Incidence of Dementia (CAIDE) score 7

Construction of LASSO-based PRS and MRS in HBCS 7

Construction of GWAMA-based PRS 8

Functional annotation of LASSO-PRS and GWAMA-PRS variants 9

Supplementary Results 10

Functional annotation results for the LASSO-PRS variants 10

Functional annotation results for the top GWAMA-PRS variants 11

Supplementary Tables 13

Table S1: Attrition analysis results in HBCS 13

Table S2: Attrition analysis results in PREDO 13

Table S3: Attrition analysis results in GLAKU 14

Table S4: Classifications of metabolic measures and transformations applied to attain minimum skewness 14

Table S5: Metabolic measures selected in metabolome-wide LASSO 14
and their associations with health-related outcomes in previous research 14
Table S6: Genome-wide association results for the variants selected by LASSO 14
Table S7: Genome-wide meta-analysis (GWAMA) results for the top GWAMA-PRS variants 15
Due to their large size, the following tables are provided in the Supplementary Excel file. 16
Table S8: Replication of LASSO-PRS variants in previous research 16
Table S9: CADD scores and eQTL mapped genes for LASSO-PRS variants 16
Table S10: Linkage of genes mapped by LASSO-PRS variants to previous research 16
Table S11: Gene-set enrichment results for LASSO-PRS variants 16
Table S12: Replication of top GWAMA-PRS variants in previous research 16
Table S13: CADD scores and eQTL mapped genes for top GWAMA-PRS variants 16
Table S14: Linkage of genes mapped by top GWAMA-PRS variants to previous research 16
Table S15: Gene-set enrichment results for top GWAMA-PRS variants 16
Table S16: Comparisons between nested regression models in the two independent HBCS-derived datasets, one including (*n* = 104) and the other excluding (*n* = 101) three stroke cases 16
Table S17: Prediction of episodic memory with adult-derived LASSO-PRS and GWAMA-PRS in PREDO 16
Table S18: Prediction of episodic memory with adult-derived LASSO-PRS and GWAMA-PRS in GLAKU 16
Supplementary Figures 17
References 19

Cohort Descriptions

Helsinki Birth Cohort Study (HBCS)

The Helsinki Birth Cohort Study (HBCS)(1, 2) consists of 13,345 individuals who were born in Helsinki in 1934-1944. For a subset of 8,760 individuals, there are data available based on birth records, child welfare clinic records, school health care records and national Finnish registers. From this subset, 2003 randomly selected individuals participated in clinical examinations in 2001-2004. A subsample of 1099 participated in the second follow-up between 2006-2011, during which episodic memory was assessed using the Consortium to Establish a Registry for Alzheimer's Disease (CERAD) test battery. Of these, 841 donated fasting blood for assessment of metabolome profile between 2006-2008 before their CERAD assessment, and 905 had data on genome-wide genotypes of the whole blood donated in 2001-2004. Out of these samples, 810 had data on both the Cardiovascular Risk Factors, Aging and Incidence of Dementia (CAIDE) score and metabolome, 846 had data on CAIDE-score, population structure (indicated with first three multidimensional scaling [MDS] components) and genome, and 655 had data on CAIDE-score, genome, and metabolome.

Prediction and Prevention of Preeclampsia and Intrauterine Growth Restriction (PREDO)

The Prediction and Prevention of Preeclampsia and Intrauterine Growth Restriction (PREDO) study is a prospective, multicenter study of Finnish women who were pregnant between 2005 and 2010 and their liveborn children. PREDO recruited 1079 women with a singleton pregnancy with a known risk factor status for preeclampsia and intrauterine growth restriction(3). The sample was appended by 89 pregnant women who were recruited independently of their risk factor status, resulting in a sample of 1168 pregnant women. The women were recruited when they arrived for their first ultrasound screening at 12 + 0 to

13 + 6 gestational weeks + days, which were conducted at ten study hospitals in Southern and Eastern Finland. Data on episodic memory assessment from the NEPSY Second Edition (NEPSY-II)(4), along with covariates used in the present study—child sex, age at episodic memory assessment and maternal education—were available for 443 children, of whom 430 also had data on genome-wide genotype data. In PREDO, for the current study, maternal education was classified as 1) primary or secondary, 2) lower tertiary or 3) upper tertiary.

Glycyrrhizin in Licorice Study (GLAKU)

The adolescents of the GLAKU (Glycyrrhizin in Licorice) cohort came from an urban community-based cohort comprising 1049 infants born between March and November 1998 in Helsinki, Finland(5). In 2009–2011, initial cohort members who had given permission to be contacted and whose addresses were traceable (*N* = 920, 87.7% of the original cohort in 1998) were invited to a follow-up, of which 692 (75.2%) could be contacted by phone (mothers of the adolescents). Of them, 451 (65.2% of those who could be contacted by phone, 49% of the invited) participated in a follow-up at a mean age of 12.3 years (SD = 0.5, range 11.0–13.2 years). Of this sample, genome-wide genotype data were available for 352 participants. Among them, episodic memory tests from the NEPSY Second Edition (NEPSY-II)(4) and the current study covariates—child sex, age at episodic memory assessment and maternal education—were available for 309 (Memory for Names) and 307 (Narrative Memory) participants. In GLAKU, for the current study, maternal education was classified as having 1) upper secondary or lower, 2) post-secondary or bachelor’s degree, 3) or master's or doctoral degree.

Supplementary Methods

Genotyping, Quality Control and Imputation

Quality control on the genotype data in HBCS

For HBCS, DNA was extracted from blood samples and genotyping was performed with the modified Illumina 610k chip by the Wellcome Trust Sanger Institute, Cambridge, UK. From the variants, only those with call rate > 0.98, HWE p value > 1×10^{-6} and minor allele count (MAC) > 3 were included, resulting in 540,506 variants. Per-sample QC included samples that whose genotype missingness < 0.05 and heterozygosity rate was within $\pm\ 4 *$ standard deviations from the population mean, leaving 1664 samples. The missing genotypes were imputed with the SISu v3 reference panel and Beagle 4.1 resulting in 16,962,023 (16,402,900 autosomal) genetic variants. For the current study, non-autosomal variants and variants with missing or duplicate IDs, or imputation quality ≤ 0.85 were excluded, resulting in 13,420,867 remaining variants.

Quality control on the genotype data in PREDO

In PREDO, fetal cord blood samples were collected according to standard procedures. DNA was extracted at the National Institute for Health and Welfare, Helsinki, Finland, and the Finnish Institute of Molecular Medicine, University of Helsinki, Finland. Genotyping was performed on Illumina Human Omni Express Exome Arrays (Illumina Inc, San Diego, CA) containing 964,193 SNPs and imputed at the Institute of Molecular Medicine Finland (FIMM) with IMPUTE 2.3.2 and Eagle v2.3 against Finnish specific SISu v2 reference panel (GRCh37) comprising 2690 high coverage whole-genome and 5093 high-coverage whole-exome sequences. Before imputation, variants with call rate < 0.95, MAF > 0.35, minor allele count < 19 or HWE p value < 1 x 10e-6 were excluded. Samples were excluded based on call rate < 0.95, heterozygosity F < 0.1, sex mismatch, and relatedness. Population outliers were

excluded based on visual inspection. There were 15,544,584 variants after imputation, including 14,986,236 autosomal variants.

Quality control on the genotype data in GLAKU

For GLAKU, DNA was extracted from blood samples (n = 80) and saliva samples (n = 277) donated at the 2009-2011 follow-up and genotyping was performed with the Illumina OmniExpress Exome 1.2 bead chip at the Tartu University, Estonia in September 2014 according to the standard protocols. Genomic coverage was extended by imputation using the 1000 Genomes Phase I integrated variant set (v3 / April 2012; NCBI build 37 / hg19) as the reference sample and IMPUTE2 software. Before imputing the following quality control filters were applied: SNP clustering probability for each genotype > 95%, call rate > 95% individuals and markers (99% for markers with minor allele frequency [MAF] < 5%), MAF > 1%, HWE p value > 1×10^{-6}. Moreover, checks on heterozygosity, sex mismatch, and relatedness were performed, and any discrepancies removed (n = 2).

Quantification of metabolomics in HBCS

Fasting blood samples were stored as serum at -80°C. A high-throughput nuclear magnetic resonance (NMR) metabolomics(6) platform (Nightingale, Helsinki, Finland) provided simultaneous quantification of 138 metabolic measures belonging to 11 groups, including amino acids, apolipoproteins, cholesterol, fatty acids and saturation, fluid balance, glycerides and phospholipids, glycolysis related metabolites, inflammation, ketone bodies, lipoprotein particle sizes and lipoprotein concentration. Glutamine was excluded due to a high percentage (63.3%) of missing data (for the other metabolic measures the percentage ranged from 0 to 18.5 %). All the remaining 137 metabolic measures were standardized to mean of 0 and SD of 1. We used the k-nearest neighbors method with k = 33 to impute metabolic measures with missing data. Metabolic measures with absolute value of skewness > 1 were

transformed with appropriate method to attain minimum skewness. See full names and classifications of metabolic measures, and applied transformations in Supplementary Table S4.

Cardiovascular Risk Factors, Aging and Incidence of Dementia (CAIDE) score

As suggested previously(7), we calculated the CAIDE-score in the HBCS as follows: age (< 47 years: 0 points, 47-53 years: 3 points, > 53 years: 4 points), sex (men: 1 point), education (≥ 10 years: 0 points, 7-9 years: 2 points, 0-6 years: 3 points), hypertension (> 140 mmHg: 2 points), obesity (> 30 kg/m^2: 2 points), hypercholesterolemia (> 6.5 mmol/L: 2 points), and physical inactivity (inactivity: 1 point). Higher score indicated more dementia risk factors.

Construction of LASSO-based PRS and MRS in HBCS

There were 846 HBCS participants with data on WLM, CAIDE and imputed genotypes, and 810 with data on WLM, CAIDE and metabolomic profiles. For both LASSO analyses, as a common test sample, we randomly selected 104 participants (12.3% and 12.8% out of n = 846 and 810, respectively) who had the CAIDE-score, and genome and metabolome data available. The remaining non-test samples having either genome (n = 742) or metabolome data (n = 706), were used in fitting the genome- and metabolome-wide LASSO models. Both genome- and metabolome-wide LASSO were adjusted for CAIDE and age at episodic memory assessment. In addition, genome-wide LASSO was adjusted for the first three components of multi-dimensional scaling (MDS) of genetic ancestry, and metabolome-wide LASSO was adjusted for the time interval (in days) between blood donation for metabolome profiling and episodic memory assessment. Since LASSO assumes predictors in the non-test and test data to be on the same scale, in the non-test data, predictors (except for genetic variants) were standardized (mean = 0 and SD = 1), while in the test-data the same predictors were scaled using the original means and SDs of the non-test data. The genetic variants were not scaled in either the non-test or test data, and their values were always equal to 0, 1 or 2.

We applied the R package implementation snpnet(8) of Batch Screening Iterative LASSO (BASIL)(8) to analyze genome-wide associations with WLM, with default settings (variants with MAF < 0.001 excluded). As suggested in the documentation, we split the 742 non-test HBCS samples randomly to training (*n* = 635) and validation (*n* = 107) sets, and then, fit the model in the training set and obtained the optimal regularization parameter corresponding to the maximum R^2 in the validation set. Then, to make most use of the validation set, we merged the training and validation sets and refitted the optimal model in the non-test samples (*n* = 742) and selected variants with a non-zero effect size for the final polygenic risk score ('LASSO-PRS').

We used the cv.glmnet function from the R package glmnet(9) to tune the LASSO regularization parameter via 4-fold-cross-validation and to select metabolome-wide associations with episodic memory in the 706 HBCS non-test samples. Metabolic measures with non-zero effect sizes in the optimal model were chosen for the final metabolic risk score ('MRS').

Construction of GWAMA-based PRS

Lahti et al. 2022(10) conducted a GWAMA for episodic memory (verbal learning with immediate recall) in > 18-year-old adults from 24 cohorts (*N* = 30,673, including HBCS) within the Cohorts for Heart and Aging Research in Genomic Epidemiology (CHARGE) consortium. In the current study, we recomputed the meta-analysis after excluding HBCS (remaining *N* = 29,785). Then we constructed a GWAMA-based PRS ('GWAMA-PRS') in the HBCS test datasets and the PREDO and GLAKU cohorts with the PRS-CS tool that applies Bayesian framework to infer posterior SNP effect sizes under continuous shrinkage priors(11). To account for linkage disequilibrium (LD) patterns in the ancestry of GWAMA, we used the 1000 Genomes Project phase 3 European LD reference panel including 1,120,696 genetic variants. As PRS-CS considers only variants shared between the LD

reference panel, GWAMA summary statistics and validation cohort, out of 5,367,331 genetic variants analyzed in GWAMA, 1,083,016 (20.2%) for HBCS, 1,097,319 (20.4%) for GLAKU and 1,082,409 (20.2%) for PREDO were included in the respective GWAMA-PRSs. To identify top variants included in the GWAMA-PRSs, we filtered variants with meta-analysis p value < 5×10^{-8}.

Functional annotation of LASSO-PRS and GWAMA-PRS variants

We used snpXplorer AnnotateMe tool(12) to annotate the LASSO-PRS and top GWAMA-PRS variants. Each variant was mapped to gene(s) with the following hierarchy: 1) If the variant was annotated as coding according to the Combined Annotation Dependent Depletion (CADD, v1.3) score, the gene affected by the coding change was assigned. 2) If the variant was non-coding, expression-quantitative-trait-loci (eQTL) mapping was performed with the GTEx v8 database across all tissues. 3) If no eQTLs were identified, the variant was mapped to the nearest gene within a 50-500kb range from the variant, incrementing by 50kb, as identified in RefSeq v98. The tool permitted a single variant to be mapped to multiple genes, and hence as a multiple testing correction, a sampling framework with 500 iterations was applied. In each iteration, one gene was randomly selected from the pool of genes associated with each variant. This set of sampled genes was then queried in the GWAS-Catalog database v1.0.2(13), and for each trait, genes overlapping between the gene query and database were obtained. Potentially deleterious variants were identified with CADD > 12. We also conducted gene-set enrichment analysis in the Gene-Ontology (GO, default in snpXplorer) pathways. There snpXplorer used again otherwise similar sampling framework as in variant-gene mapping but tested associations with each term. Enriched terms with p value < 0.05 were considered statistically significant.

Supplementary Results

Functional annotation results for the LASSO-PRS variants

Based on the snpXplorer output, the current genome-wide LASSO replicated association of the intergenic variant rs6813517 (4q32.3) which has previously been significantly associated (p value = 3×10^{-8}) with episodic memory assessed with word list delayed recall test from the CERAD test battery(14) in a genome-wide meta-analysis in Debette et al. 2015(15). However, this replication was not independent of HBCS since HBCS was included as a discovery cohort in their meta-analysis. There were also three other variants previously linked to different traits, although not related to cognition (Supplementary Table S8).

Out of all 274 LASSO-PRS variants, one was mapped by direct gene coding region, 118 based on eQTL GTEx tissue expression, 143 based on positional mapping, and 12 were not mapped (Fig. S1C). Variants were distributed across all autosomal chromosomes (Fig. S1 A and B). Number of genes mapped per variant ranged from 1 to 10, and 166 variants mapped exactly one gene (Fig. S1A). Altogether, 310 different genes were mapped (Supplementary Tables S9 and S10). The sole variant located in a coding region was the synonymous SNP rs17810074, on chromosome 15 in DENND4A (Supplementary Table S9), a gene linked with intelligence(16) (Supplementary Table S10). A total of 13 variants satisfied the CADD > 12 threshold, indicating potential deleteriousness (Supplementary Table S9).

Altogether, 177 different genes were identified through eQTL mapping, and gene expression associated with mapped variants was detected in 49 different tissues (Supplementary Table S9). For the brain tissues (n = 13), 43 variants were identified as significant eQTLs, corresponding to 35 eQTL-mapped genes. For example, rs6709706, rs6706041, rs6709337 and rs4662705—all located in chromosome 2—were significant eQTLs for BIN1 in brain cerebellar hemisphere and brain cerebellum (Supplementary Table S9). BIN1 has previously

been linked to variants associated with diagnosis and family history of Alzheimer's Disease (AD)(17) (Supplementary Table S10). Variants rs12916923 and rs12913203, both located in chromosome 15, were significant eQTLs for PWRN1 in several brain tissues (Supplementary Table S9). PWRN1 has previously been associated with ratio of total tau to amyloid β_{42}(18), a biomarker for AD (Supplementary Table S10).

Gene-set enrichment analysis revealed six pathways, including neuron differentiation, generation of neurons and nervous system development, to be enriched with the mapped genes (Supplementary Table S11).

Functional annotation results for the top GWAMA-PRS variants

Due to large amount (> 1 million) of variants included in each cohort's GWAMA-PRS, we restricted to the top variants (n = 12) with meta-analysis p value $< 5 \times 10^{-8}$. Based on the functional annotation results, the regulatory region variant rs4420638 (p value = 3.91×10^{-10}) at 19q13.32 has previously replicated significantly (all p values < 5×10^{-8}) for cognition-related outcomes in multiple studies independent of HBCS: it has been reported as a risk variant for AD(19-23) and cognitive impairment(24). Consistent with these findings, it has also been associated with age of AD onset(19, 25), several biomarkers for AD(24, 26-29) and cognitive function(30) and psychomotor performance(31) (Supplementary Table S12). In addition, in the non-independent genome-wide meta-analyses in Debette et al. 2015(15), which included HBCS, rs4420638 was significantly associated (p values < 5×10^{-8}) with verbal declarative memory based on both paragraph delayed recall alone and on combined tests of verbal delayed recall. Based on snpXplorer, 5 additional variants have been previously associated with non-cognition-related traits. The remaining 6 variants showed no prior trait associations (Supplementary Table S12).

All the 12 top GWAMA-PRS variants were mapped based on their eQTL GTEx tissue expression (Fig. S2 C). Number of genes mapped per variant ranged from 2 to 21 (Fig. S2 A). All the mapped genes were exclusively located in chromosomes 3 and 19 (Fig. S2 B). In total, 25 genes were identified through eQTL mapping, and expression was observed in 41 different tissues (Supplementary Table S13). Variant rs4420638 was an eQTL for APOE and APOC1 (Supplementary Table S13), which both have been linked for example with diagnosis and family history of AD(17) and cognitive impairment(24) (Supplementary Table S14). Therefore, it was surprising that rs4420638 was the only variant not associated with gene expression in brain tissues. Instead, the other 11 variants—all located at 3p21.1—were significant eQTLs for 10 different genes expressed in 10 different brain tissues (Supplementary Table S13). Three variants had CADD > 12, indicating potential deleteriousness (Supplementary Table S13). Gene-set enrichment analysis revealed 3 statistically significant enriched pathways but none of them related to nervous system development or function. (Supplementary Table S15).

Supplementary Tables

Table S1: Attrition analysis results in HBCS

	**Included (*N* = 1001)**	**Excluded (*N* = 1002)**	***p* value**
CAIDE	7.0 (2.0)	7.8 (2.3)	0.019
Age, years	68.5 (2.8)	68.4 (3.1)	0.741
Education, years	12.5 (3.7)	12.0 (3.6)	0.01
Males, n, (%)	425 (42.5%)	503 (50.2%)	0.001
Systolic blood pressure, mm Hg	143.8 (19.6)	147.1 (20.6)	0.0002
BMI, kg/m^2	27.3 (4.3)	28.0 (5.0)	0.001
Total cholesterol, mmol/L	5.9 (1.0)	6.0 (1.1)	0.197
Physically inactive, n, (%)	69 (6.9%)	72 (7.5%)	0.693

Second and third columns provide mean (SD), unless otherwise indicated, in individuals who were and were not in the current study, respectively, from the total HBCS sample of 2003 individuals. To test the difference between the included and excluded samples, Pearson's Chi-squared test was applied for sex and physical inactivity, and two-sided t-test was applied for the other predictors. Resulting *p* value provided in the fourth column.

Table S2: Attrition analysis results in PREDO

	**Included (*N* = 443)**	**Excluded (*N* = 725)**	***p* value**
Age, years	8.6 (0.9)	9.1 (1.1)	0.002
Males, n (%)	221 (49.9%)	388 (55.2%)	0.091
Maternal education level: primary or secondary, lower tertiary, upper tertiary; n (%)	160 (36.1%), 112 (25.3%), 171 (38.6%)	352 (50.9%), 157 (22.7%), 182 (26.3%)	1.43×10^{-6}

Second and third columns provide mean (SD) for child age at episodic memory assessment, and frequencies (and proportions) for the child sex and maternal education in individuals who were and were not in the current study, respectively, from the total PREDO sample of 1168 individuals. To test the difference between the included and excluded samples, two-sided t-test was applied for child age at episodic memory assessment, and Pearson's Chi-squared test was applied for sex and maternal education. Resulting *p* value provided in the fourth column.

Table S3: Attrition analysis results in GLAKU

	Included (*N* = 309)	Excluded (*N* = 611)	*p* value
Age, years	11.9 (0.6)	11.9 (0.6)	0.920
Males, n (%)	146 (47.2%)	317 (51.9%)	0.209
Maternal education level: ≤ upper secondary, post-secondary or bachelor’s degree, master’s degree or doctoral dissertation; n (%)	50 (16.2%), 147 (47.6%), 112 (36.2%)	15 (15.8%), 45 (47.4%), 35 (36.8%)	0.993

Second and third columns provide mean (SD) for child age at episodic memory assessment, and frequencies (and proportions) for the child sex and maternal education in individuals who were and were not in the current study, respectively, from the total GLAKU sample of 920 individuals. To test the difference between the included and excluded samples, two-sided t-test was applied for child age at episodic memory assessment, and Pearson's Chi-squared test was applied for sex and maternal education. Resulting *p* value provided in the fourth column.

Table S4: Classifications of metabolic measures and transformations applied to attain minimum skewness

Due to large size of the table, it is presented in the Supplemental Excel file.

Table S5: Metabolic measures selected in metabolome-wide LASSO and their associations with health-related outcomes in previous research

The first four columns provide abbreviation, full name, classifications and effect sizes of the metabolic measures determined in metabolome-wide LASSO in the non-test sample of HBCS (n = 706). The other columns provide information on replications in previous research. Due to large size of the table, it is presented in the Supplemental Excel file.

Table S6: Genome-wide association results for the variants selected by LASSO

Due to large size of the table, it is presented in the Supplemental Excel file.

Table S7: Genome-wide meta-analysis (GWAMA) results for the top GWAMA-PRS variants

RSID	CHR	Position	Effect.allele	Non.effect.allele	PRS-CS.Effect.size	GWAMA.P.value	Effect.allele.frequency
rs6445528	3	52572447	G	A	0.03098742	6.336E-09	0.476562
rs1133415	3	52575831	A	G	0.02689778	8.237E-09	0.476262
rs1961958	3	52585990	G	A	0.02374293	1.477E-08	0.406851
rs6778844	3	52596398	C	T	0.03088697	1.747E-08	0.40625
rs2289249	3	52597664	A	G	0.02980349	2.241E-08	0.40625
rs12487445	3	52618319	C	A	0.01851202	1.84E-08	0.40625
rs11177	3	52721305	A	G	0.02577315	2.135E-08	0.40655
rs6976	3	52728804	T	C	0.02991482	3.499E-08	0.40655
rs3774354	3	52817675	A	G	0.00802889	6.814E-09	0.365385
rs2239551	3	52818579	A	G	0.01927474	4.391E-08	0.39393
rs2286798	3	52821177	C	A	0.01762597	3.906E-08	0.366587
rs4420638	19	45422946	G	A	-0.09243364	3.908E-10	0.282151

The table provides RSID, genomic coordinates, effect and non-effect alleles, PRS-CS effect sizes, meta-analysis p values and effect allele frequencies in HBCS for the GWAMA-PRS variants with meta-analysis p value < 5×10^{-8}.

Due to their large size, the following tables are provided in the Supplementary Excel file.

Table S8: Replication of LASSO-PRS variants in previous research

Table S9: CADD scores and eQTL mapped genes for LASSO-PRS variants

Table S10: Linkage of genes mapped by LASSO-PRS variants to previous research

Table S11: Gene-set enrichment results for LASSO-PRS variants

Table S12: Replication of top GWAMA-PRS variants in previous research

Table S13: CADD scores and eQTL mapped genes for top GWAMA-PRS variants

Table S14: Linkage of genes mapped by top GWAMA-PRS variants to previous research

Table S15: Gene-set enrichment results for top GWAMA-PRS variants

Table S16: Comparisons between nested regression models in the two independent HBCS-derived datasets, one including (n = 104) and the other excluding (n = 101) three stroke cases

Table S17: Prediction of episodic memory with adult-derived LASSO-PRS and GWAMA-PRS in PREDO

Table S18: Prediction of episodic memory with adult-derived LASSO-PRS and GWAMA-PRS in GLAKU

Supplementary Figures

Figure S1: Functional annotation results visualized for 262 (excluding 12 variants that snpXplorer was not able to map) LASSO-PRS variants. Number of genes mapped per variant in A), and per chromosome in B). In C) annotation method (direct coding region, eQTL or positional) of each variant is indicated.

A)

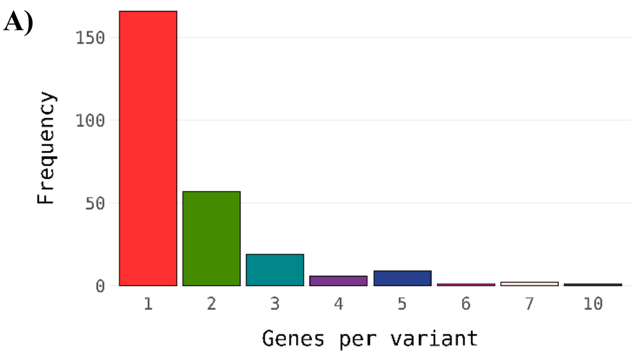

B)

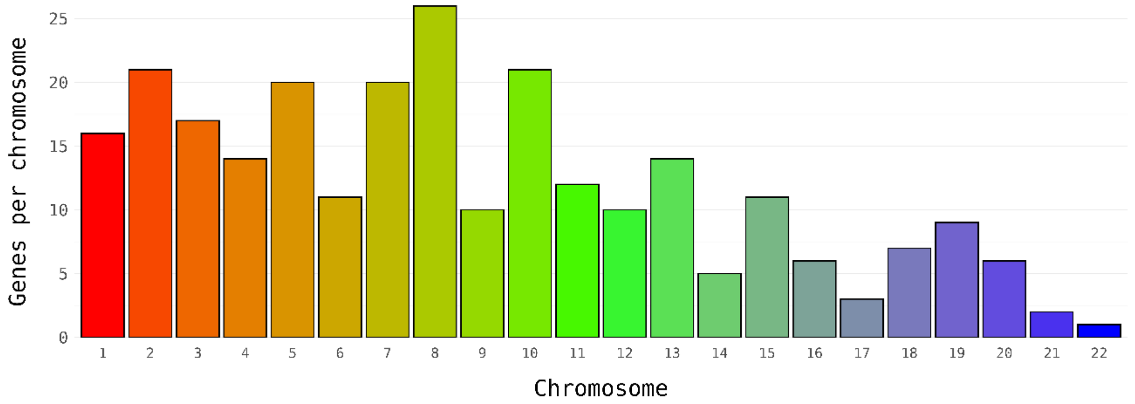

C)

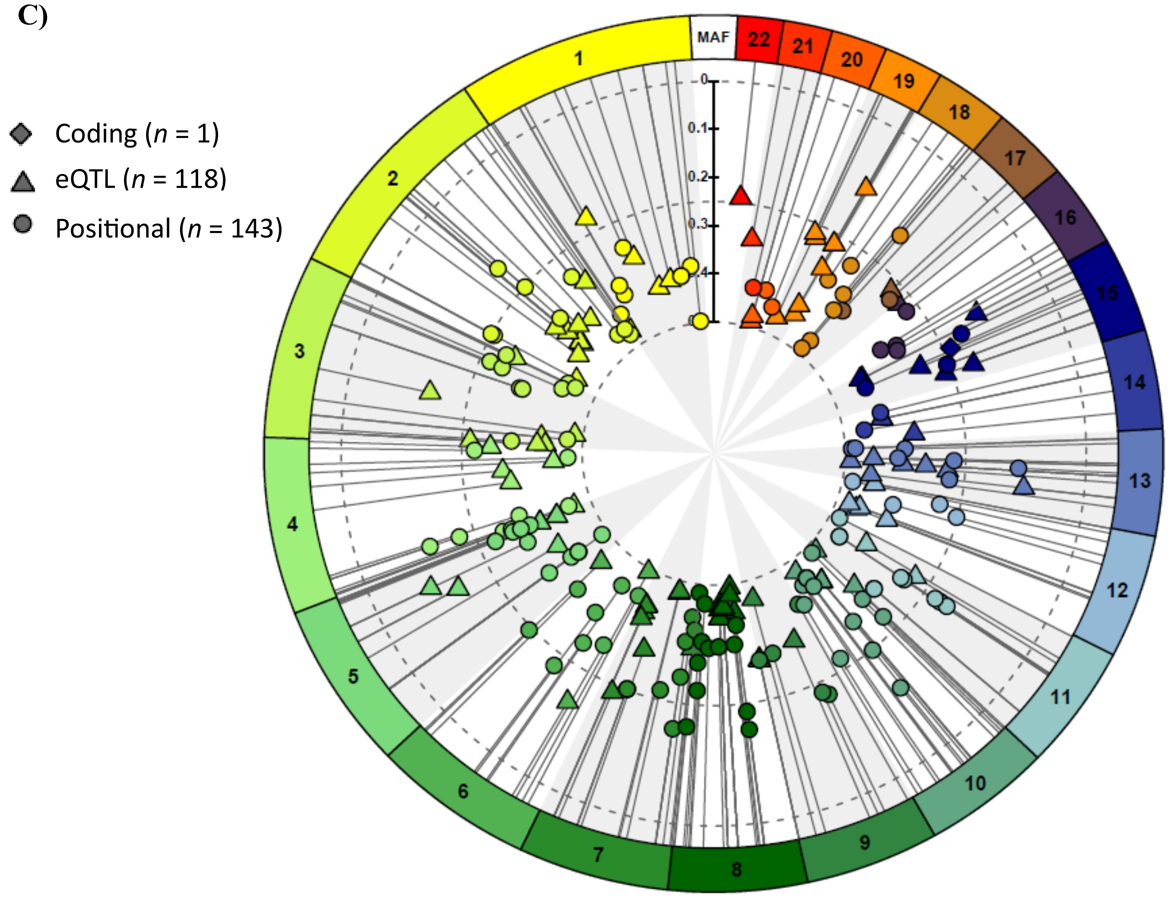

Figure S2: Functional annotation results visualized for the 12 top GWAMA-PRS variants. Number of genes mapped per variant in A), and per chromosome in B). In C) annotation method (direct coding region, eQTL or positional) of each variant is indicated. All the 12 variants were annotated via eQTL-mapping.

A

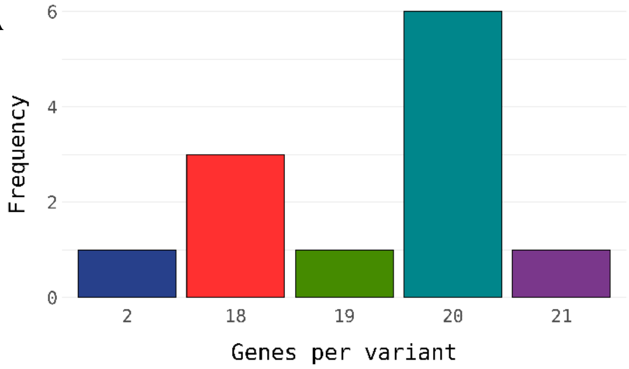

B)

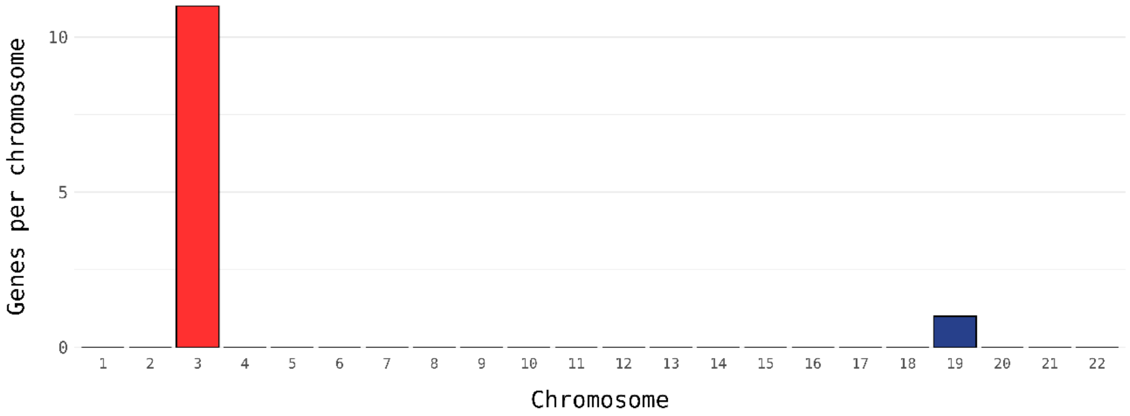

C)

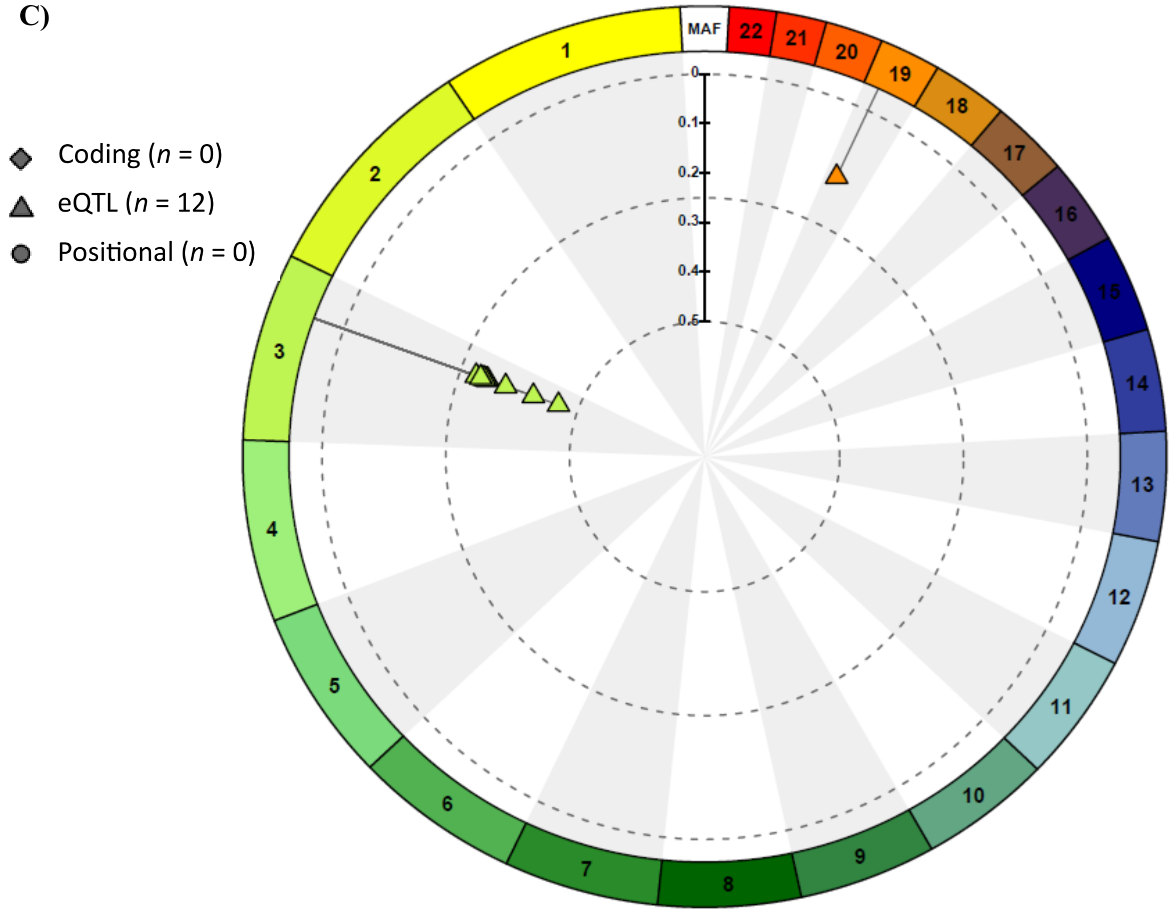

References

1. Barker DJ, Osmond C, Forsén TJ, Kajantie E, Eriksson JG. Trajectories of growth among children who have coronary events as adults. N Engl J Med. 2005;353(17):1802-9.

2. Eriksson JG, Guzzardi MA, Iozzo P, Kajantie E, Kautiainen H, Salonen MK. Higher serum phenylalanine concentration is associated with more rapid telomere shortening in men. Am J Clin Nutr. 2017;105(1):144-50.

3. Girchenko P, Lahti M, Tuovinen S, Savolainen K, Lahti J, Binder EB, et al. Cohort Profile: Prediction and prevention of preeclampsia and intrauterine growth restriction (PREDO) study. International Journal of Epidemiology. 2017;46(5):1380-+.

4. Korkman M, Kirk, U., Kemp S. L. NEPSY-II: Second edition – Administrative Manual. San Antonio, TX: Harcourt Assessment; 2007.

5. Strandberg TE, Jarvenpaa AL, Vanhanen H, McKeigue PM. Birth outcome in relation to licorice consumption during pregnancy. American journal of epidemiology. 2001;153(11):1085-8.

6. Soininen P, Kangas AJ, Wurtz P, Tukiainen T, Tynkkynen T, Laatikainen R, et al. High-throughput serum NMR metabonomics for cost-effective holistic studies on systemic metabolism. Analyst. 2009;134(9):1781-5.

7. Kivipelto M, Ngandu T, Laatikainen T, Winblad B, Soininen H, Tuomilehto J. Risk score for the prediction of dementia risk in 20 years among middle aged people: a longitudinal, population-based study. The Lancet Neurology. 2006;5(9):735-41.

8. Qian J, Tanigawa Y, Du W, Aguirre M, Chang C, Tibshirani R, et al. A fast and scalable framework for large-scale and ultrahigh-dimensional sparse regression with application to the UK Biobank. PLoS genetics. 2020;16(10):e1009141.

9. Friedman J, Hastie T, Tibshirani R. Regularization Paths for Generalized Linear Models via Coordinate Descent. J Stat Softw. 2010;33(1):1-22.

10. Lahti J, Tuominen S, Yang Q, Pergola G, Ahmad S, Amin N, et al. Genome-wide meta-analyses reveal novel loci for verbal short-term memory and learning. Molecular psychiatry. 2022;27(11):4419-31.

11. Ge T, Chen CY, Ni Y, Feng YA, Smoller JW. Polygenic prediction via Bayesian regression and continuous shrinkage priors. Nature communications. 2019;10(1):1776.

12. Tesi N, van der Lee S, Hulsman M, Holstege H, Reinders MJT. snpXplorer: a web application to explore human SNP-associations and annotate SNP-sets. Nucleic acids research. 2021;49(W1):W603-W12.

13. NHGRI-EBI GWAS Catalog v1.0.2.

14. Morris JC, Heyman A, Mohs RC, Hughes JP, van Belle G, Fillenbaum G, et al. The Consortium to Establish a Registry for Alzheimer's Disease (CERAD). Part I. Clinical and neuropsychological assessment of Alzheimer's disease. Neurology. 1989;39(9):1159-65.

15. Debette S, Verbaas CAI, Bressler J, Schuur M, Smith A, Bis JC, et al. Genome-wide Studies of Verbal Declarative Memory in Nondemented Older People: The Cohorts for Heart and Aging Research in Genomic Epidemiology Consortium. Biological psychiatry. 2015;77(8):749-63.

16. Davies G, Lam M, Harris SE, Trampush JW, Luciano M, Hill WD, et al. Study of 300,486 individuals identifies 148 independent genetic loci influencing general cognitive function. Nature communications. 2018;9.

17. Schwartzentruber J, Cooper S, Liu JZ, Barrio-Hernandez I, Bello E, Kumasaka N, et al. Genome-wide meta-analysis, fine-mapping and integrative prioritization implicate new Alzheimer's disease risk genes. Nat Genet. 2021;53(3):392-402.

18. Maxwell TJ, Corcoran C, Del-Aguila JL, Budde JP, Deming Y, Cruchaga C, et al. Genome-wide association study for variants that modulate relationships between

cerebrospinal fluid amyloid-beta 42, tau, and p-tau levels. Alzheimers Res Ther. 2018;10(1):86.

19. Herold C, Hooli BV, Mullin K, Liu T, Roehr JT, Mattheisen M, et al. Family-based association analyses of imputed genotypes reveal genome-wide significant association of Alzheimer's disease with OSBPL6, PTPRG, and PDCL3. Molecular psychiatry. 2016;21(11):1608-12.

20. Coon KD, Myers AJ, Craig DW, Webster JA, Pearson JV, Lince DH, et al. A high-density whole-genome association study reveals that APOE is the major susceptibility gene for sporadic late-onset Alzheimer's disease. J Clin Psychiatry. 2007;68(4):613-8.

21. Webster JA, Myers AJ, Pearson JV, Craig DW, Hu-Lince D, Coon KD, et al. Sorl1 as an Alzheimer's disease predisposition gene? Neurodegener Dis. 2008;5(2):60-4.

22. Li H, Wetten S, Li L, St Jean PL, Upmanyu R, Surh L, et al. Candidate single-nucleotide polymorphisms from a genomewide association study of Alzheimer disease. Arch Neurol. 2008;65(1):45-53.

23. Nazarian A, Yashin AI, Kulminski AM. Genome-wide analysis of genetic predisposition to Alzheimer's disease and related sex disparities. Alzheimers Res Ther. 2019;11(1):5.

24. Liu C, Chyr J, Zhao W, Xu Y, Ji Z, Tan H, et al. Genome-Wide Association and Mechanistic Studies Indicate That Immune Response Contributes to Alzheimer's Disease Development. Front Genet. 2018;9:410.

25. Kamboh MI, Barmada MM, Demirci FY, Minster RL, Carrasquillo MM, Pankratz VS, et al. Genome-wide association analysis of age-at-onset in Alzheimer's disease. Molecular psychiatry. 2012;17(12):1340-6.

26. Li J, Zhang Q, Chen F, Meng X, Liu W, Chen D, et al. Genome-wide association and interaction studies of CSF T-tau/Abeta(42) ratio in ADNI cohort. Neurobiology of aging. 2017;57:247 e1- e8.

27. Li J, Zhang Q, Chen F, Yan J, Kim S, Wang L, et al. Genetic Interactions Explain Variance in Cingulate Amyloid Burden: An AV-45 PET Genome-Wide Association and Interaction Study in the ADNI Cohort. Biomed Res Int. 2015;2015:647389.

28. Yan Q, Nho K, Del-Aguila JL, Wang X, Risacher SL, Fan KH, et al. Genome-wide association study of brain amyloid deposition as measured by Pittsburgh Compound-B (PiB)-PET imaging. Molecular psychiatry. 2021;26(1):309-21.

29. Guo Y, Xu W, Li JQ, Ou YN, Shen XN, Huang YY, et al. Genome-wide association study of hippocampal atrophy rate in non-demented elders. Aging (Albany NY). 2019;11(22):10468-84.

30. de la Fuente J, Davies G, Grotzinger AD, Tucker-Drob EM, Deary IJ. A general dimension of genetic sharing across diverse cognitive traits inferred from molecular data. Nat Hum Behav. 2021;5(1):49-58.

31. De Jager PL, Shulman JM, Chibnik LB, Keenan BT, Raj T, Wilson RS, et al. A genome-wide scan for common variants affecting the rate of age-related cognitive decline. Neurobiology of aging. 2012;33(5):1017 e1-15.
